# Three-dimensional reconstruction of gigapixel whole-mount histopathology specimens with RAPID

**DOI:** 10.1038/s41598-026-46776-4

**Published:** 2026-04-02

**Authors:** Daan Schouten, Jeroen van der Laak, Diederik Somford, Heidi Küsters-Vandevelde, Nadieh Khalili, Geert Litjens

**Affiliations:** 1 Department of Pathology, Radboud University Medical Center, Nijmegen, the Netherlands; 2 Oncode Institute, Utrecht, the Netherlands; 3 Department of Urology, Canisius Wilhelmina Hospital, Nijmegen, The Netherlands; 4 Prosper Prostate Cancer Clinics, Nijmegen/Eindhoven, The Netherlands; 5 Department of Pathology, Canisius Wilhelmina Hospital, Nijmegen, The Netherlands

**Keywords:** Anatomy, Computational biology and bioinformatics, Engineering, Medical research

## Abstract

Histopathology often serves as the gold standard in medical diagnosis but lacks a spatial three-dimensional axis of information due to the inherent process of two-dimensional tissue slide preparation. Recovering this third dimension would enable correlation with in vivo imaging, improve multimodal data integration, and open new doors for three-dimensional quantitative tissue analysis. This study presents the RAPID framework, which tackles this task of registering whole slide images of whole-mount histopathology specimens into a three-dimensional stack. Our proposed framework leverages a DINOv2-pretrained ViT-L14 foundational model to consistently detect anatomical features, which are used to align a stack of unregistered whole slide images (WSIs) to obtain a three-dimensional reconstruction. RAPID is optimized to work with high-resolution WSIs at 0.25 $\upmu$m/pixel, leading to gigapixel reconstructions that can be used for cell-level downstream tasks. We validate our framework on various external validation sets and find that RAPID obtains an accurate reconstruction in 91.7–93.5% of cases, thereby significantly outperforming a current state-of-the-art reconstruction method. Unlike existing methods limited to serial sections, RAPID handles the sparse sampling intervals (3000–4000 $\upmu$m) routinely used in clinical pathology. We demonstrate its utility for three-dimensional radiology-pathology correlation, enabling direct volumetric comparison between histopathology and pre-operative imaging.

## Introduction

### Motivation

Histopathology is the gold standard for clinical diagnosis, given its unparalleled resolution compared to in vivo imaging modalities. However, due to the nature of preparing histopathology slides, achieving this resolution generally incurs the drawback of losing the three-dimensional relationship with the rest of the tissue specimen. Once multiple sections are cut from the specimen, prepared on a glass slide, stained, and digitised with a scanner, the original spatial relationship between sections will be lost as tissue transformations have been introduced in these processing steps. Hence, the collection of slides has, at this point, regressed towards a sparse, transformed representation of the original tissue. Moreover, each slide is a two-dimensional abstraction of a three-dimensional structure, which can hinder assessment of tissue architecture in isolation. However, reconstructing these slides into a three-dimensional volume can partially recover this lost context and better approximate the in situ anatomy. The recovery of this third dimension is particularly vital in scenarios where a pathology specimen needs to be compared to other three-dimensional data sources, such as preoperative imaging in radiology-pathology correlation studies^1–3^. Hence, obtaining a stack of two-dimensional slides registered in third dimension, hereafter called a three-dimensional reconstruction, of the original tissue specimen has excellent potential to optimize this correlation workflow, which may lead to faster and more accurate diagnosis.

Consequently, there is a growing scientific interest in three-dimensional pathology^4,5^ and three-dimensional tissue analysis^6–12^. Successful incorporation of this third dimension may prove fruitful in a wide range of applications, such as tissue visualization in a clinical setting^11,13^, extensive quantitative tissue analysis^6,8^ or the unraveling of tissue microarchitecture^7^. Methods for obtaining a three-dimensional tissue representation can be divided into nondestructive three-dimensional pathology^14^ or three-dimensional reconstruction of whole slide images (WSIs) that were acquired through a conventional destructive approach^15^. Although both methods have their merits, a significant benefit of the latter is that it uses readily available data and techniques. This contrasts with non-destructive pathology, where specialized equipment is required for data acquisition, leading to workflow modifications, staff (re)training, and increased cost. Furthermore, the ever-growing collection of archival slides can be readily leveraged to develop and validate three-dimensional reconstruction methods. Developing robust methods for three-dimensional reconstruction, however, has hitherto proven to be a non-trivial task.

### Related work

A recent systematic review that analysed three-dimensional reconstruction methods found that a modest 29 articles have been published on this topic since 2018^15^. Kiemen et al. recently proposed the CODA pipeline^6^, which integrates three-dimensional reconstruction of serially sliced specimens with cell detection and segmentation to unravel microarchitectural patterns. Although CODA can create a three-dimensional reconstruction from hundreds of tissue sections, notable limitations include i) the inability to process registered WSIs at full resolution, ii) a lack of validation on sparsely sampled tissue specimens, and iii) a significant computational overhead of 1–4 days per case. Gatenbee et al. recently introduced the VALIS algorithm^16^, which was the first method to tackle the problem of registering and saving multiple gigapixel WSIs in their original full resolution. Retaining this original resolution is a crucial component for most downstream tasks with the reconstructed sample, such as the application of deep learning-based tumour segmentation methods or three-dimensional viewing^11^. Despite VALIS’ broad validation and competitive performance on several benchmarks, it shared CODA’s limitation of lacking validation on sparsely sampled tissue specimens. Indeed, Gatenbee et al. acknowledge that registration may fail with increased distances between sections. More generally, all recent methods summarized by Kurz et al. target serial sections with $\le$200 $\upmu$m spacing^15^, far denser than typical clinical sampling (e.g., 3000–5000 $\upmu$m in prostatectomy^17^ and 4000 $\upmu$m in mastectomy^18^).

Hence, despite recent progress, current algorithms remain largely confined to research settings and often fail to generalize to clinical stacks with sparse sampling. Moreover, the gigapixel scale of WSIs makes reconstruction computationally demanding: even simple rigid transformations can be impractical in memory unless implemented efficiently (e.g., in a tile-based manner). Together, these constraints have hindered robust, full-resolution three-dimensional reconstruction in routine pathology workflows.

### Contributions

This paper postulates that current reconstruction methods do not generalize to sparsely sampled specimens due to the overfitting of local landmarks and features. Although this is understandable when reconstructing serially sectioned slides, this dramatically limits the further application of these methods in clinical scenarios where sampling is more sparse. Therefore, we propose solving the reconstruction problem in global feature space, where more general, high-level features from foundational models are used to guide the reconstruction. Our main contributions entail i) the application of a vision foundational model to guide reconstruction, ii) an optimized global feature matching method to robustly register slides with varying degrees of similarity, and iii) a highly efficient implementation for linear and deformable registration of full-resolution slides. We validate our method on a multicentre dataset with specimens sampled at 5 to 4000 $\upmu$m intervals to demonstrate its generalizability for various applications. We have made our algorithm publicly available at https://github.com/computationalpathologygroup/rapid and provide access to a full-resolution sample case for reproducibility of our results.

## Methods

### Data

Data to train and validate the algorithm was primarily sourced from prostatectomy specimens sectioned at 4000 $\upmu$m intervals. Our method was expected to generalize to smaller rather than larger sectioning intervals more easily. We collected one private prostatectomy dataset for model development and externally validated our model on a range of private and publicly available datasets with varying sampling intervals and organs. For the model development set, we retrospectively included 142 patients who underwent robot-assisted radical prostatectomy between 2018-2022 in the Canisius Wilhelmina Ziekenhuis (Nijmegen, the Netherlands). After prostatectomy, the specimen was fixated in 10% buffered formalin for 24 hours. Sectioning of the specimen was performed free-hand, where the lab technician aimed to create equally distanced cuts from base to apex at an interval of 4000 $\upmu$m. Tissue blocks were subsequently processed to create whole-mount sections and were stained with hematoxylin and eosin (H&E). All slides were digitized on a 3DHISTECH P1000 scanner (Budapest, Hungary) with a 40$\times$ objective at 0.25 $\upmu$m/pixel resolution. This model development set was further subdivided on a patient level into a training (n=58 patients) and validation (n=38 patients) set to tune algorithm hyperparameters, and an independent test set (n=46 patients) was used for an unbiased performance estimate. An internally developed quality control tool was employed to verify that WSIs did not contain significant scanning artefacts. When major scanning artefacts (i.e., partially scanned slides or large out-of-focus areas) were detected, the slide was rescanned until sufficient quality was obtained. In addition, cases where a slide in the middle of the stack was missing, leading to an 8000 $\upmu$m sampling interval between the involved slides, were excluded (n=3). Finally, specimen ink markings were utilized to manually verify that a slide had not been horizontally flipped during the slide preparation process, as this would prevent a correct reconstruction. If a horizontal flip was encountered upon inspection, specialized software was used to flip the WSI back to its proper state, congruent in orientation with the other WSIs.

In addition to the internal validation, we externally validated our algorithm on a prostatectomy dataset (n=48) from the Radboud University Medical Centre (Nijmegen, the Netherlands). Similar to the model development set, this dataset contained whole-mount prostatectomy slides sampled at a 4000 $\upmu$m interval and followed the exact specifics in slide preparation, scanning, and quality control. Furthermore, we validated our algorithm on two publicly available datasets. First, the dataset from^19^ consists of 28 prostatectomy specimens sparsely sampled at 3000-4000 $\upmu$m intervals. Since this dataset primarily comprises pseudo-whole-mount sections obtained with HistoStitcher^20^, we aim to verify whether our method is robust against stitching artefacts. Since this dataset was not created for three-dimensional reconstruction purposes, we select all cases (n=14) that adhere to the requirement above that adjacent slides must have a similar sampling interval. Second, we externally validate our method on the dataset of^21^, as this dataset has previously been used as a benchmark for several three-dimensional reconstruction methods. This dataset consists of two components, namely i) a murine prostate serially sectioned in 260 slides at a 5 $\upmu$m interval and ii) a murine liver serially sectioned in 47 slides at a 5 $\upmu$m interval. A unique factor of this dataset is the presence of annotated landmarks, which enables the computation of a target registration error (TRE) as a metric for reconstruction accuracy. Although the landmarks for the prostate specimen entail manual annotation of corresponding visual features (i.e., split cells) between adjacent slides, the landmarks in the liver specimen consist of a laser-induced hole present in all slides. Therefore, the liver dataset presents an interesting opportunity to measure the reconstruction accuracy at varying sampling intervals since this interval can be modified using one out of every *n* slides for the reconstruction. A summary of all datasets and their characteristics is provided in Table 1.

**Table 1:** Overview of datasets used for model development and validation. The number of whole slide images per case is reported as mean (range).

Partition	Origin	Tissue	Cases	WSIs per case	Sampling method	Sampling interval
Training	CWZ	Human prostate	58	5 (4–8)	Sparse	4000 $\upmu$m
Validation	CWZ	Human prostate	38	5 (4–7)	Sparse	4000 $\upmu$m
Internal Testing	CWZ	Human prostate	46	5 (4–8)	Sparse	4000 $\upmu$m
External Testing	Radboudumc	Human prostate	48	7 (4–9)	Sparse	4000 $\upmu$m
External Testing	^21^	Murine liver	1	47	Serial	5 $\upmu$m
External Testing	^21^	Murine prostate	1	260	Serial	5 $\upmu$m
External Testing	^19^	Human prostate	14	5 (3–9)	Sparse	3000-4000 $\upmu$m

### RAPID framework

**Fig. 1:**
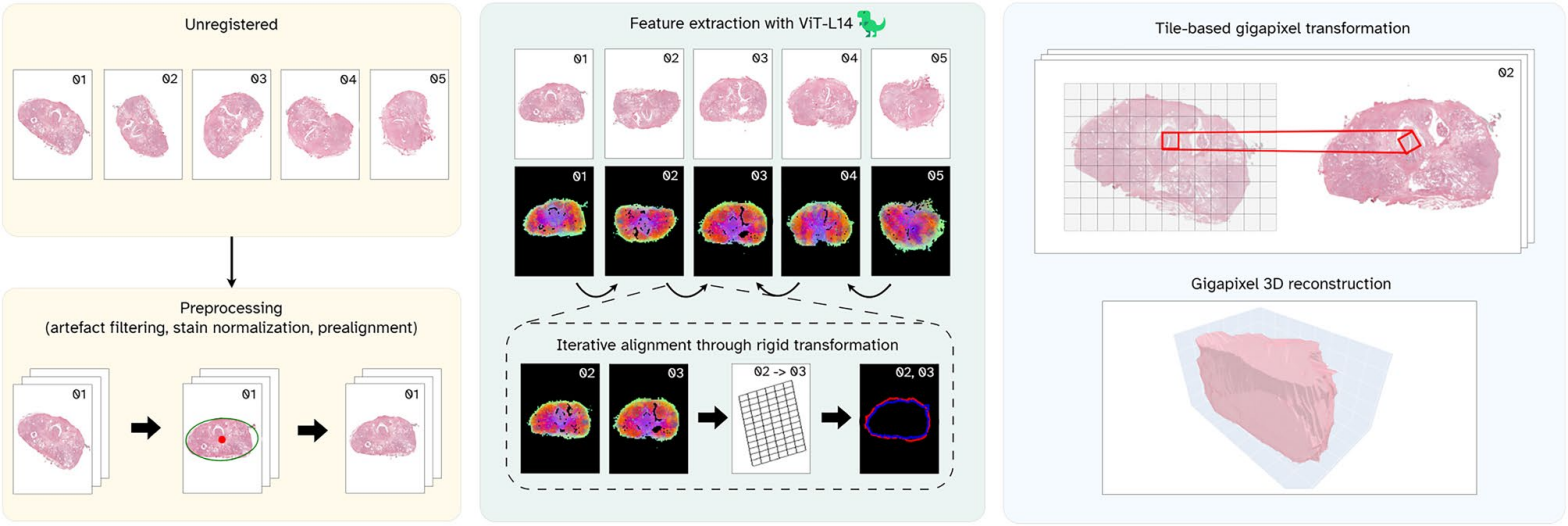
Schematic overview of the RAPID framework, consisting of three distinct components. The left panel visualizes the preprocessing of a stack of unregistered WSIs, the middle panel visualizes the global feature extraction with the iterative pairwise alignment, and the right panel demonstrates the tile-based approach for full-resolution reconstruction.

In this section, we introduce the different components of our framework, ’Reconstruct Any Pathology In 3 Dimensions’ (RAPID). The RAPID framework entails multiple components schematically visualized in Figure 1. Each component will be more extensively described in the respective subparagraph. Pseudo-code for RAPID is provided in Algorithm 1.

#### Preprocessing

RAPID employs a dedicated preprocessing pipeline to harmonize all WSIs before attempting the reconstruction. First, a tissue foreground mask is generated using Otsu thresholding^22^. The slide part not categorized as foreground will be masked to erase any background noise or minor artefacts that could influence the reconstruction result. Second, we employ Reinhard’s algorithm^23^ for stain normalization to reduce staining variability throughout the stack. Specifically, we compute a median colour matrix for all WSIs and map all WSIs to this common colour space. Third, we downsample all WSIs to a common resolution (i.e. 20 $\upmu$m/pixel) that brings the image size closest to $2000\times 2000$ pixels and apply padding such that all WSIs have the same square dimensions. Note that, despite using a computationally efficient downsampled version of the WSI to perform all upcoming reconstruction steps, these transformations are parametrized to linearly scale with resolution, enabling the generation of the reconstructed stack at the exact resolution as the initial WSIs. In contrast to other state-of-the-art reconstruction algorithms^6,16^, we do not convert the slides to a greyscale representation but propose preserving the full RGB colour space.

#### Global features

Before elaborating on the exact implementation, we first introduce the concept of global features. In our reconstruction task, we consider global features to be a coarse anatomical and structural representation of a given WSI. Although sparsely sampled adjacent WSIs are not expected to have the same landmarks (e.g., tumour outlines or smaller glands), we expect their global features to be similar. Hence, optimizing the alignment between global features of adjacent WSIs is likely a reasonable proxy for aligning anatomical structures of said WSIs. Extraction of global features is generally accomplished by applying a foundational model that was pretrained in a self-supervised manner, often with the DINOv2 paradigm^24^. In this study, we employ a vision transformer model (ViT-L14) pretrained on the LVD-142M dataset with DINOv2 and do not further finetune it on domain data. After extraction of global features from two WSIs, the matching can be learned by training a simple neural network to optimize their similarity^25^, although several dedicated frameworks exist that have been extensively validated^26,27^. Here, we focus on the state-of-the-art RoMa framework^26^, which was proposed for the natural image domain to estimate the homography matrix for images of the same object under extreme viewpoint and illumination changes. Although several frameworks exist^27^, RoMa stands out for its strong reliance on global features from a foundational model for alignment, with the additional incorporation of finer features for alignment finetuning. We postulate that our scenario of WSIs with slightly different tissue macro architectures may be analogous to extreme viewpoint changes in the natural image domain. We expect that RoMa may solve this reconstruction task by focusing on the global features of the WSIs.

#### Reconstruction initialization

The reconstruction is initialized with an initial estimate of the rigid transformation for each WSI. This is achieved by fitting an enclosing ellipse through the tissue foreground mask to determine each WSI’s centre point and rotation. The middle WSI in the stack is then selected as the reference image, and all WSIs are mapped to a standard coordinate system where each WSI’s centre point and rotation align with that of the reference image. Although this initialization step generally filters out most rotation discrepancies between adjacent slides, a mismatch of 180 degrees may still be present in some cases due to the ellipsoid’s symmetric nature. We hypothesized that this 180-degree rotation could be detected between adjacent slides when comparing global features extracted with a vision transformer (ViT-L14) pretrained with the DINOv2 paradigm^24^. Specifically, it was expected that global features of two adjacent WSIs would have a higher cosine similarity when images were correctly aligned than when there was a 180-degree rotation mismatch. Hence, this initialization step is concluded by iterating over image pairs in the entire stack and applying a 180-degree rotation to an image when it improves the cosine similarity with the adjacent slide.

#### Reconstruction finetuning

After the initialization step, all WSIs are roughly aligned in the same coordinate space but lack a finer registration of global features. We, therefore, estimate a rigid transformation to align global features between adjacent WSIs further. Specifically, we employ the RoMa framework^26^ described earlier. For this step, the global features are combined with finer features obtained through a VGG19 network^28^ pretrained on ImageNet, to form a feature pyramid of the image. This feature pyramid contains image features at different spatial resolutions and although this pyramid could theoretically be constructed through feature extraction at different network depths from a single encoder, the original RoMa paper demonstrated that such a single encoder setup was suboptimal. Hence, a ViT-L14 and VGG19 model are employed to generate the coarse and fine features, respectively. As we do not modify the feature pyramid setup from the original RoMa implementation, we refer the reader to the original RoMa paper for further details^26^. We note that, similar to the global feature extraction step, this finer feature extraction step is performed on the low-resolution version of the WSI for computational efficiency. We then aim to find matches in the feature pyramids of the two images, which can then be used to estimate a transform that would spatially align these features. Although methods in the natural image domain generally aim to find the homography matrix that aligns two images, we limit ourselves to a rigid transform to ensure anatomically feasible transformations. We explicitly omit the scaling and/or shearing factor commonly present in affine transformations, as true size differences between adjacent slides near the extremities of the reconstructed specimen are anatomically viable and should thus not be corrected. In addition, we employ the RANSAC algorithm^29^ to estimate a set of inliers based on an empirical threshold and base the rigid transform only on these inliers rather than on all matching features. Aligning the entire stack of WSIs is then achieved by iterative pairwise registration where both ends are aligned towards the frame of reference. In other words, if $k$ is the total number of slides and $r$ is the index of the reference frame, $r+1$ is first aligned to $r$ after which $r+2$ is aligned to the previously aligned version of $r+1$. This is then repeated for the alignment of slides $r-n-1$ to $r-n$ and $r+n+1$ to $r+n$ until all $k$ slides have been registered. After obtaining the optimal rigid transform for each WSI, an optional deformable registration using thin-plate splines is employed to optimize the reconstruction locally. This additional deformable registration is reserved for use cases with denser sampling intervals and smaller distances between adjacent slides.

Finally, the transformations obtained from the downsampled images are propagated to the highest resolution level in each WSI, enabling a reconstruction at the original resolution of the initial WSIs. This is achieved by efficiently implementing rigid and deformable registration, where the transformation parameters scale linearly with the different resolution levels. Specifically, in the case of deformable registration with thin-plate splines, the Cartesian grid on which the splines are represented can be linearly scaled to any resolution level without recomputing the optimal transform, enabling efficient deformable registration of the full-resolution slide. Furthermore, the rigid and deformable transforms are executed tile-based using the Pyvips library^30^ since applying any transformation to a WSI directly is computationally infeasible. Notably, the deformable transform is represented as a dense backward coordinate map, where each output pixel independently stores the source coordinate to sample from, making the mapping decomposable into independent tiles without boundary artifacts. With this tile-based approach, the WSI is divided into small tiles, after which the desired transform is applied to each tile, effectively streaming the transformed tiles to their respective locations in the transformed slide. Since this streaming approach limits computational requirements to several tiles rather than the whole image, WSIs of arbitrary size can be transformed on regular clinical workstations, stimulating the clinical applicability of the proposed reconstruction method.

**Algorithm 1:**
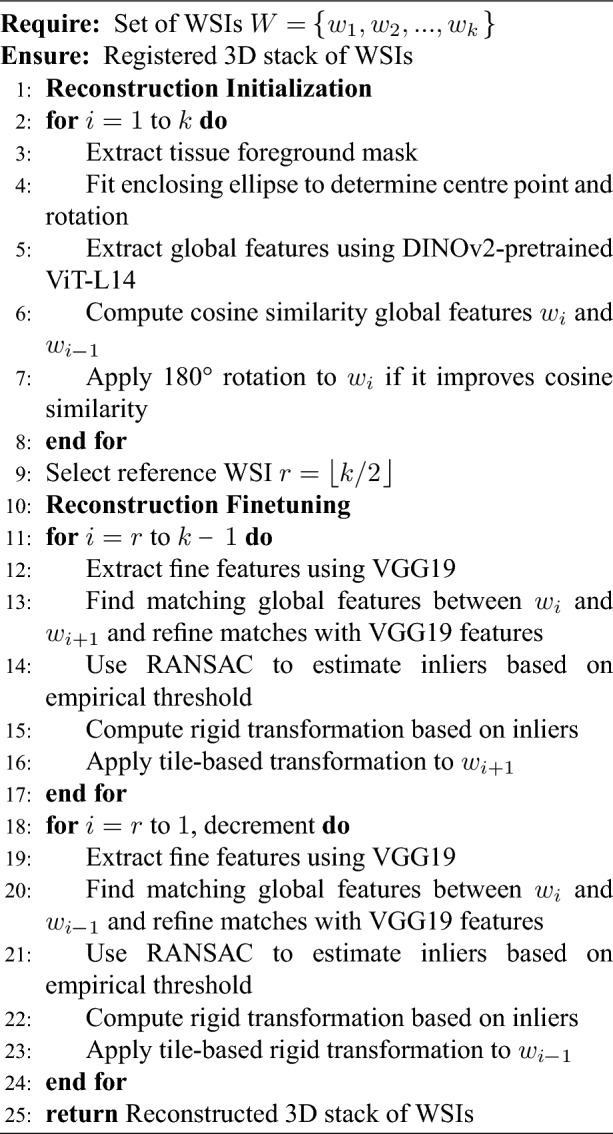
RAPID pseudo-code.

#### Evaluation

Evaluating the accuracy and robustness of three-dimensional reconstruction methods is non-trivial since there is no ground truth, and multiple satisfactory solutions can coexist. To assess the multiple aspects of an accurate solution, we introduce three metrics that quantitatively capture different components of the reconstruction result. First, we introduce the general reconstruction accuracy, which captures the general success of achieving a meaningful reconstruction from WSIs that may have vastly different orientations. A case is considered to have a successful reconstruction when, compared to the reference WSI, all other WSIs differ at most 15 degrees in their rotation. This ground truth rotation was obtained by manually annotating all WSIs for a given case, considering the specimen’s original anatomical shape. The threshold of 15 degrees was chosen empirically to balance the requirement for visually smooth reconstructions while considering the considerable intra-observer variability of this annotation task. The general reconstruction accuracy for a dataset is then defined as the percentage of the total $n$ cases where all $k$ slides of a given case $i$ adhere to this heuristic.

Second, we introduce an overlap metric that captures the overlap $O$ between all $k-1$ pairs of adjacent slides for a given case $i$. Since tissue size differences between adjacent slides can occur naturally and should not be penalized or corrected, the smallest fragment’s area is the denominator. The overlap now captures the extent by which the smallest slide overlaps with the larger one. Let $A_n$ and $A_{n+1}$ be the area of each slide in a pair of adjacent slides, then the overlap $O$ for a case with $k$ slides and $k-1$ pairs of adjacent slides is defined as:

1 $$\begin{aligned} \text {O} = \frac{1}{k-1} \sum _{n=1}^{k-1} \frac{A_n \cap A_{n+1}}{\min (A_n, A_{n+1})} \end{aligned}$$

Third, the average target registration error (TRE) for corresponding global features in adjacent slides is introduced to assess the reconstruction result on a slightly more local level. Global feature matches representing anatomical areas are detected for each pair of adjacent slides, and the Euclidean distance between locations of corresponding features is computed. To improve robustness against outliers, the median TRE of all matches from a pair of slides is determined, after which the average TRE over all pairs is computed to capture the TRE over the entire three-dimensional reconstruction result of a given case. Let $F_{n,i}$ and $F_{n+1, i}$ be the coordinates of matched anatomical feature pair $i$ of adjacent slides $n$ and $n+1$, respectively, then the $TRE$ for a case with $k$ slides, $k - 1$ pairs of adjacent slides and $m$ corresponding feature pairs is defined as:

2 $$\begin{aligned} \text {TRE} = \frac{1}{k-1} \sum _{n=1}^{k-1} \text {median} \left( \left\| F_{n,i} - F_{n+1,i} \right\| \right) \nonumber \\ \quad \text {for} \; i = 1, 2, \ldots , m \end{aligned}$$

The three aforementioned metrics are reported for each dataset to demonstrate RAPID’s performance on various tasks. We compare the performance of the proposed method against the baseline method VALIS^16^. We use the default parameters for benchmarking VALIS, which have been thoroughly validated in the respective paper^16^. We use the same parameters for our proposed method for each dataset, and limit RAPID to only the rigid registration component without additional deformable registration steps. All benchmarks were run on a Linux machine with an Intel Xeon e5-2630 CPU, an NVIDIA GeForce RTX 2080 Ti graphics card, and 40 GB RAM.

### Statistical analysis

To assess whether RAPID significantly outperforms VALIS, we performed paired statistical tests for each metric and dataset. Since both methods were applied to the same cases, we obtained paired observations enabling case-by-case comparison. For the binary accuracy metric, we employed McNemar’s exact test, which is specifically designed for paired nominal data and focuses on discordant pairs where the two methods have discordant reconstruction outcomes (i.e. RAPID obtained an accurate reconstruction whereas VALIS might not). For the continuous metrics overlap and TRE, we first assessed normality of the pairwise differences using the Shapiro-Wilk test. When normality could not be rejected at $\alpha = 0.05$, a paired t-test was performed. Otherwise, the non-parametric Wilcoxon signed-rank test was used. To control for multiple testing within each cohort, we applied the Holm-Bonferroni correction across the three metrics, which provides family-wise error rate control.

### Ethics statement

The Institutional Review Board (IRB) of Radboud University Medical Center, the METC Oost-Nederland (Nijmegen, the Netherlands), approved the use of anonymized data for training and validating the algorithm. Since all data was acquired retrospectively and fully anonymized, the IRB (The Institutional Review Board (IRB) of Radboud University Medical Center, the METC Oost-Nederland) waived the need for informed consent for this study (2022–15,878). Furthermore, this research was performed following the Declaration of Helsinki.

## Results

### Global features

**Fig. 2:**
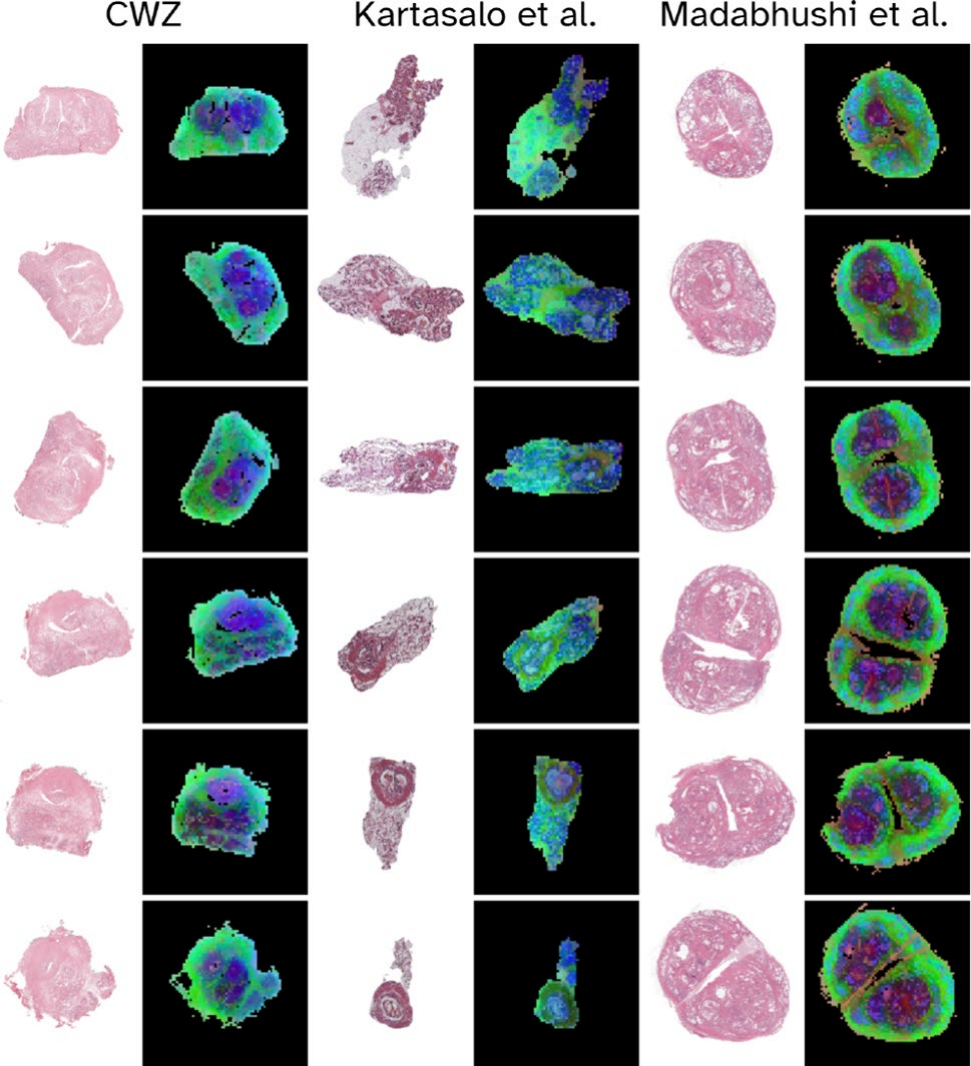
Visualization of the first three principal components of ViT-L14 features extracted from three cases from our external test sets. Columns show a prostatectomy sample from the external Radboud set (left), a prostate sample from the external Kartasalo et al. set (middle) and a prostatectomy sample from the external Madabhushi et al. set (right).

To demonstrate the robustness of DINOv2-pretrained ViT-L14 features for global feature matching throughout the stack of WSIs, we visualize these features for three different examples each taken from a different test set. Specifically, we downsample each WSI to $1036\times 1036$ to fit the ViT-L14 architecture and interpolate the positional embeddings of each patch to handle a higher resolution than the $518\times 518$-sized images on which the model was trained. This $1036\times 1036$ resolution was empirically found to deliver an optimal balance between reconstruction accuracy (typically associated with higher resolution) and computational efficiency (typically associated with lower resolution). Next, we randomly rotate each image by a number between 0 and 360 degrees to demonstrate that these global features are relatively rotation invariant and thus suitable for three-dimensional reconstruction tasks where images are not guaranteed to have the same rough orientation. This image is fed through the network, after which the features from the final layer are extracted, resulting in a $74\times 74\times 1024$ feature vector. This feature vector thus represents the combined 1024-dimensional embedding for each $14\times 14$ patch from the original image. To visualize all features for a given stack of images, we perform a principal component analysis on the concatenated feature vector of all images, after which we extract the three principal components for each feature vector. Consequently, this results in *n* $74\times 74\times 3$ feature vectors for *n* images, which can be visualized as RGB images, as shown in Figure 2. A noteworthy observation in the example from the Madabhushi et al. dataset (right two columns) is that these global features seem to correspond with larger anatomical areas, such as the benign prostate hyperplasia nodules visualized in shapes of purple in the feature representation. Conversely, comparatively minor variations in tissue architecture in the peripheral zone are represented in a more uniform colour in the feature representation map. As such, a similar high-level tissue architecture of adjacent WSIs seems to lead to visually similar feature representations, which may subsequently be leveraged to acquire three-dimensional reconstructions based on the general tissue architecture.

### Baseline comparison

We compare our method against VALIS^16^, a strong WSI registration and three-dimensional reconstruction baseline. We use all the default parameters for VALIS, but limit the transformation to a rigid transformation without scaling component. Since preliminary testing on cases with larger slice distances revealed that VALIS attempted implausible scaling corrections and/or deformable registration, we limit the algorithm to a basic rigid transform for a fair comparison. In a comparison on computational overhead for both methods we find marginal differences in both wall-clock time and computational requirements. We note that the wall-clock time is practically exclusively determined by the size and resolution of the WSIs, given that actual reconstruction time is modest at 1 and 2 minutes per case for VALIS and RAPID, respectively. For the average prostatectomy case in our cohort with 5 WSIs of 4–6 GB each (approximately 150k $\times$ 100k pixels), applying the reconstruction and writing the entire stack of WSIs at full resolution (0.25 $\upmu$m/pixel) to disk required 3–4 hours per case for both VALIS and RAPID. However, performing the same reconstruction with downsampled WSIs (approximately 6k $\times$ 4k pixels) resulted in an average wall-clock time of 3 minutes for VALIS and 4 minutes for RAPID to perform the reconstruction and write the registered stack to disk. Regardless of the resolution of the input WSIs, both methods proved to be memory-efficient at 2 GB peak RAM for VALIS and 4 GB peak RAM for RAPID, with RAPID requiring an additional 6 GB of VRAM to extract global and local features using the ViT-L14 and VGG19 model, respectively.

### Slice distance experiments

**Fig. 3:**
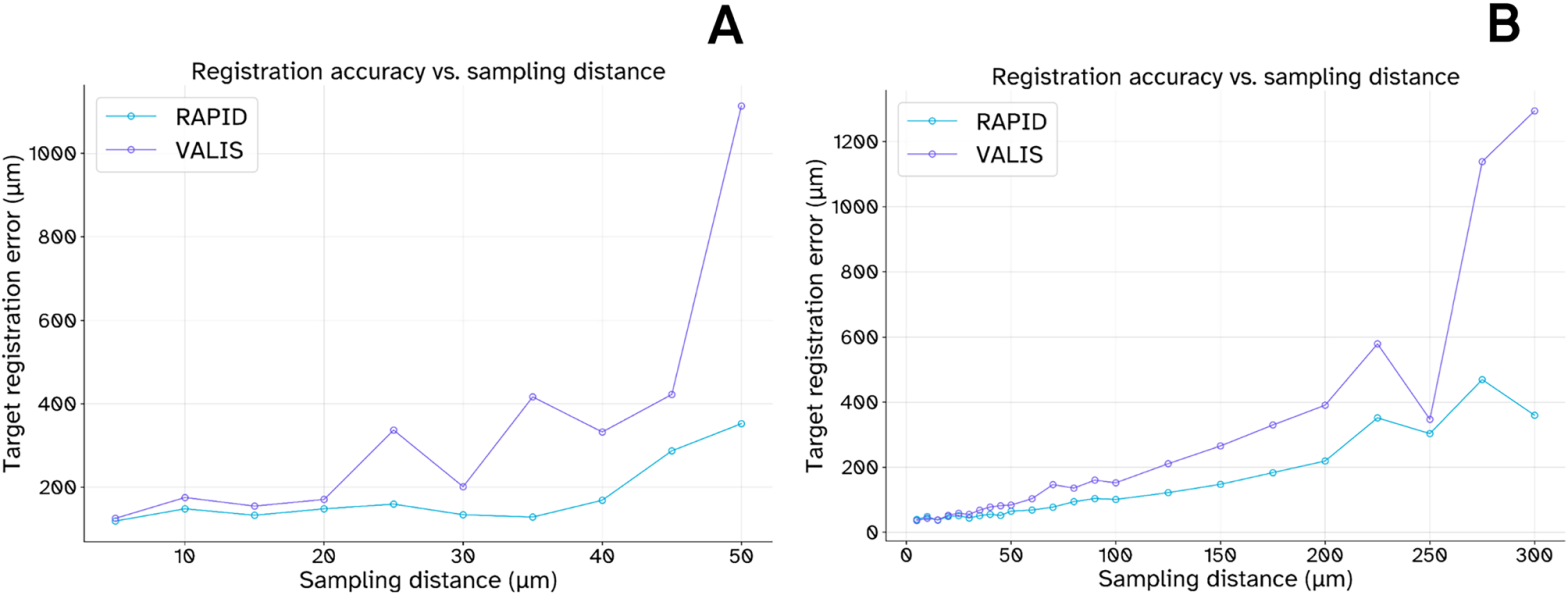
Target registration error for various sampling distances in a serially sectioned murine liver (**A**) and a serially sectioned murine prostate (**B**). Note that the TRE in (**A**) was computed based on ground truth landmarks and the TRE in (**B**) was estimated based on distance between global features used for reconstruction.

We employ the previously described dataset from^21^ to study the effect of slice distance on reconstruction accuracy. Since the serially sliced murine liver in this dataset was punctured with four laser beams, which were later annotated, we can utilize these landmarks to compute the TRE. We simulate an increasing slice distance by performing the reconstruction with one out of every *n* WSIs where we let *n* range from 1 to 10, resulting in a 5–50 $\upmu$m slice distance. For simulations where we sample at most one out of every three slides, we repeat this experiment three times with different WSIs to ensure consistency in our findings. We compute the TRE for a given simulation by first averaging the TRE of the four individual landmarks per adjacent slide pair, and subsequently averaging the TRE per slide pair to obtain a TRE for the entire reconstruction. We average the TRE across experiments for the slice distances where we perform repeated experiments to get one representative value for each slice distance. The effect of this increasing slice distance on TRE is visualized in Figure 3A, demonstrating that RAPID and VALIS perform on par for smaller slice distances. Still, RAPID shows improved robustness for larger slice distances. We repeat this experiment for the serially sectioned murine prostate, where we again sample one out of every *n* WSIs where we let *n* range from 1 to 60, resulting in a 5–300 $\upmu$m slice distance. Again, for simulations where we sample at most one in three slides, we repeat the experiment three times with three different selections of WSIs with the same slice distance. In contrast to the liver specimen, this prostate specimen does not have ground truth landmarks that can be used to compute the TRE. Therefore, we instead leverage RAPID’s ability to estimate the TRE based on the distance between global features that were used during reconstruction. Although VALIS has a similar capability to estimate TRE, both methods use markedly different features in their reconstruction and using the estimated TRE of both methods would not result in an apples-to-apples comparison. Therefore, for each sampling experiment, we feed the WSIs registered by VALIS back into RAPID to estimate the TRE without introducing any additional transformations, enabling a fair head-to-head comparison. We use the exact same TRE computation method described for the liver specimen, where we first compute a mean TRE per slide pair, which is then averaged over the entire reconstruction. The effect of the increasing slice distance on estimated TRE is visualized in Figure 3B, corroborating our findings from the experiments on the murine liver. Again, we observe similar performance for smaller sampling distances but a diverging performance gap for increasingly sparse sampling.

### Prostatectomy reconstructions

**Fig. 4:**
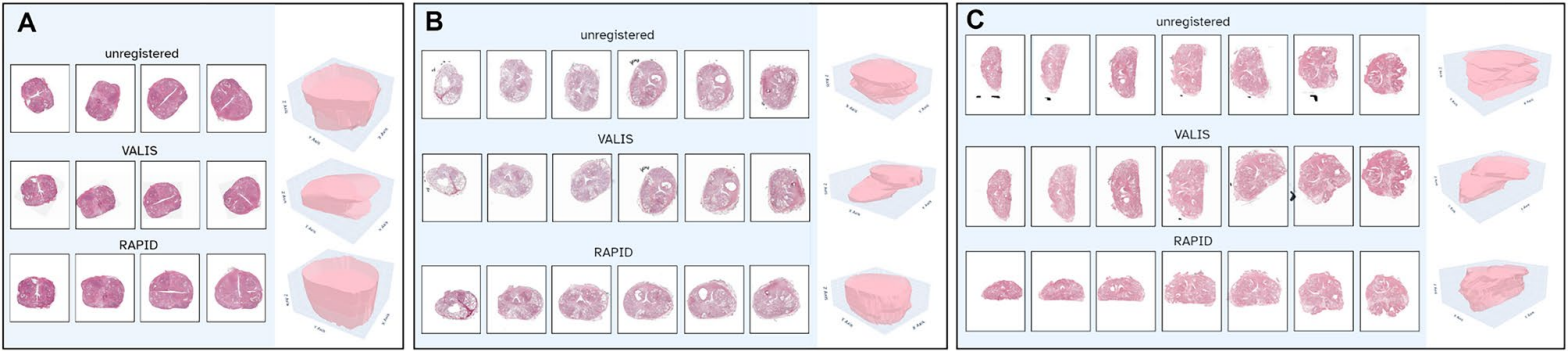
Visualization of a representative reconstruction from each test set (A -^19^, B - CWZ, C - Radboud). For each pane, the top row displays the original unregistered stack and the middle and bottom row display the reconstruction results from VALIS and RAPID, respectively. Additionally, a 3D visualization of the registered stack demonstrates the smoothness of the reconstructed result.

Although the previous experiment investigated RAPID’s performance in various simulated sampling intervals, most clinical settings employ a sampling interval that is an order of magnitude greater than the 300 $\upmu$m investigated for the murine prostate. To verify that RAPID generalizes to clinically relevant sampling intervals, we further validated RAPID on a multicentre prostatectomy dataset. We do not finetune any parameters for these datasets but treat this as an independent test set where RAPID’s parameters remain unchanged. We further test the robustness of our method against varying initial conditions by randomly rotating each WSI for a given reconstruction case by a random number between 0 and 360 degrees. Again, we compare our method against VALIS with the same settings mentioned earlier and parse the reconstructions from VALIS through RAPID to enable a head-to-head comparison of the estimated TRE. Table 2 summarizes the main reconstruction accuracy metrics for the internal testing cohort (CWZ) and external testing cohorts (Radboud and^19^). RAPID outperforms VALIS even more strongly in this clinical setting where sampling intervals are even greater. Although we observe that VALIS occasionally obtains a reasonable reconstruction of several adjacent slides, it seems to lack general robustness, as indicated by the comparatively low number of cases where all WSIs had a similar (i.e., < 15-degree difference) orientation. This is further exemplified in Figure 4, demonstrating a representative reconstruction example for each test set reconstructed by both VALIS and RAPID. An interesting observation from this figure is that RAPID correctly identifies that the two most right WSIs in panel C contain a part of the seminal vesicles in the bottom of the WSI, which should not be matched to any prostate tissue. This observation is even more striking when the full stack is visualized in three dimensions, which is accomplished via through-plane linear interpolation of two adjacent images in the stack and binarizing the image in tissue vs. background to create a contiguous three-dimensional representation of the specimen. Indeed, this three-dimensional visualization displays a slight protrusion of these vesicles, concordant with the original in situ situation. In addition, these three-dimensional visualizations elegantly demonstrate the in situ shape of the prostate, with a relatively flat dorsal side and a more convex anterior aspect.

**Table 2:** Quantitative comparison of reconstruction accuracy metrics across three prostatectomy datasets. Accuracy refers to the collective orientation accuracy of all WSIs in the reconstruction as defined in subsection 2.2.5. Overlap refers to the metric in equation (1) and the TRE, expressed in mm, refers to equation (2). Overlap and TRE are expressed in mean and standard deviation for each cohort. Numbers highlighted in bold indicate the highest performance. Asterisks indicate Holm–Bonferroni-corrected statistical significance of RAPID versus VALIS ($$^*p<0.05$$, $$^{**}p<0.01$$, $$^{***}p<0.001$$).

Method	CWZ (n=46)			Radboud (n=48)			Madabhushi et al. (n=14)		
	Accuracy $\uparrow$	Overlap $\uparrow$	TRE $\downarrow$	Accuracy $\uparrow$	Overlap $\uparrow$	TRE $\downarrow$	Accuracy $\uparrow$	Overlap $\uparrow$	TRE $\downarrow$
VALIS^16^	29.5%	0.87 ± 0.066	6.25 ± 2.94	36.2%	0.87 ± 0.059	5.34 ± 3.27	25.0%	0.83 ± 0.10	2.25 ± 1.50
RAPID (ours)	93.5%^***^	0.98 ± 0.012^***^	1.27 ± 0.71^***^	91.7%^***^	0.96 ± 0.013^***^	1.14 ± 0.47^***^	92.9%^*^	0.97 ± 0.015^**^	0.74 ± 0.33^*^

### Multimodal validation

**Fig. 5:**
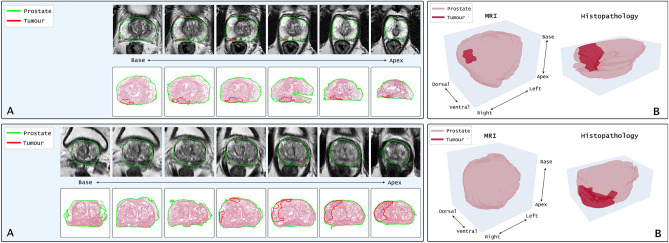
Multimodal visualization of two representative cases from the external Radboud prostatectomy cohort. Each row depicts a unique patient, with panel A showing side-by-side 2D visualizations of slices from the pre-operative MRI and 2D slices from the 3D reconstruction obtained through RAPID. Panel B displays an interpolated 3D render of the prostate gland and tumour volume for both the MRI and the 3D reconstruction.

To demonstrate the downstream utility of the 3D reconstructions facilitated by RAPID, we visualize two representative cases from the external prostatectomy cohort alongside their corresponding pre-operative MRI in Figure 5. Both patients appeared in the PI-CAI public training set^31^, which provided an automatically generated binary mask of the prostate gland and an expert manual voxel-level annotation of clinically significant prostate cancer (csPCa), defined as Gleason grade group $\ge$ 2. An expert uropathologist with 20 years of experience manually annotated the extent of csPCa on the original whole slide images, which were subsequently registered in 3D using RAPID, enabling a 3D visualization of the prostate gland with the contained lesion. This 3D reconstruction obtained through RAPID now enables a direct 3D-3D comparison of tumour manifestation on both modalities, without the need for expensive research-oriented workflows such as 3D printed molds^32^. We do not perform any additional MRI-histopathology registration^33^, since the current comparison already yields important clinical takeaways and further multimodal registration is out-of-scope for the current study.

First, the top row of Figure 5 demonstrates a case with a suspected PIRADS 4 lesion in the right-dorsal aspect of the peripheral zone, which proved to be a Gleason 4+3 lesion upon performing an in-bore MRI-guided biopsy. The radical prostatectomy performed 5 months after the initial MRI revealed a Gleason 3+4 pattern, as visualized in the non-registered 2D-2D comparison in panel A. A striking observation is the apparent difference in tumour size, which is best appreciated on the side-by-side 3D visualization of both modalities in panel B, demonstrating a clear elongated growth pattern along the basal-apical axis. Although direct volume comparisons should be interpreted with care due to tissue deformations occurring in the slide preparation process^17^ and interpolation artefacts due to limited resolution in the basal-apical axis, it remains notable that the lesion volume estimate amounted to 0.37 ml on MRI compared to 1.53 ml, a fourfold increase, on the 3D histopathology reconstruction. Indeed, it has been established that MRI is prone to underestimating the extent of prostate cancer relative to measurements on histopathology^34,35^. With the 3D reconstructions enabled by RAPID, this effect can be studied on a greater scale as RAPID does not require dedicated sectioning protocols but instead operates on archival data. Furthermore, although out-of-scope for the current study, RAPID paves the way for 3D-3D multimodal registration, enabling voxel-level comparison of tumour behaviour in different modalities.

The bottom row of Figure 5 visualizes a case with a suspected PIRADS 4 lesion in the right-ventral aspect of the transition zone, with MRI-guided biopsy confirming a Gleason 3+3 lesion. Due to a notably elevated PSA of 33 ng/ml (PSA density 0.67), a radical prostatectomy was performed 3 months after the diagnostic MRI, revealing a Gleason 4+3 lesion as shown in panel A. For this case, however, a side-by-side comparison of lesion size/volume was infeasible as non-csPCa (Gleason grade group = 1) is typically not annotated on MRI. Indeed, contemporary prostate cancer detection models are often solely focussed on detecting csPCa, treating non-csPCa as the background label to prevent overdiagnosis and overtreatment^31^. However, for this particular case, there exists a discrepancy in cancer significance status, as the biopsy returned a Gleason 3+3 whereas the prostatectomy returned a Gleason 4+3. In other words, if one were to include this patient in a cohort to train prostate cancer detection models, one would have to choose between assigning this patient to the non-csPCa or the csPCa subset. Traditionally, the choice for ground truth label has been governed by pragmatism where the biopsy label was favored over the prostatectomy label due to the difficulty of cognitively fusing the spatial prostatectomy outcomes with the lesion location in the MRI. However, the 3D reconstruction facilitated by RAPID provides an important stepping stone for cognitive fusion of prostatectomy outcome, and streamlines the establishment of ground truth in these scenarios. Again, we note that RAPID does not perform any 3D-3D multimodal registration, but rather bridges the gap from 2D pathology to 3D pathology and thereby streamlines multimodal (3D) data integration for various use cases, among others ground truth establishment.

### Ablation analysis

To study the effect of different components of the RAPID framework, we perform extensive ablation experiments and report the performance on all three clinical validation sets to compare with our proposed method in Table 3. Unless otherwise specified, the ablation experiments use our RAPID framework’s exact same general configuration. First, we studied the effect of our proposed approach of using global features rather than detailed keypoints to perform the reconstruction. In these experiments, we retain the iterative alignment through rigid transformations but base the rigid transformation matrix on pairs of matched keypoints in adjacent WSIs. We investigated this approach for both the classical SIFT^36^ keypoint detection method with brute force matching and the recent deep learning-based LightGlue keypoint detector and matcher^37^. Although we do not obtain any meaningful reconstructions for the SIFT-based method, the LightGlue-based method can achieve some correct reconstructions, albeit at a much lower overall accuracy than our proposed approach. Additionally, we study another recent keypoint detection method, OmniGlue^27^, which uses LightGlue as a base keypoint detector and leverages DINOV2 features to guide the keypoint matching process further. Although OmniGlue generally outperforms LightGlue in the natural image domain and generalizes well to out-of-distribution scenarios, we do not observe this in our reconstruction task. Hence, these ablations demonstrate that using global features rather than localized keypoints is key for three-dimensional reconstruction from sparsely sampled specimens.

In addition to these main feature-matching approaches, we study the effect of our RAPID framework’s smaller yet influential design choices. First, we study the impact of estimating the rigid transformation based on all matching global features (RAPID-no-ransac) rather than using RANSAC^29^ to estimate this transform based on a subset of inliers. The quantitative comparison in Table 3 demonstrates that the proposed RANSAC approach delivers a small yet noticeable performance improvement. Second, we investigate the effect of correcting 180-degree rotations before estimating a rigid transformation. We compare this with a single-shot approach (RAPID-single-shot), where a rigid transformation is estimated without correcting 180-degree orientation mismatches. Although this approach is uncommon in the natural image domain, where such extreme orientation discrepancies are rare (e.g., the sky generally does not appear at the bottom of the image), this component seemed vital in the performance of our RAPID framework. This ablation shows that despite the visually similar appearance of the global features, the extreme orientation mismatch prevents a reasonable estimation of the rigid transform to align these WSIs in most cases. Lastly, we perform an ablation experiment (RAPID-overlap) to correct these 180-degree orientation mismatches by choosing the configuration with the highest overlap between adjacent WSIs rather than the highest cosine similarity of global features. Interestingly, Table 3 shows that this overlap optimization leads to a similar overlap metric and slightly lower TRE at the cost of notably lower reconstruction accuracy, indicating that no single metric can capture the success of a given method.

**Table 3:** Quantitative comparison of reconstruction accuracy metrics across three prostatectomy datasets for all ablation experiments. Numbers highlighted in bold indicate the highest performance. RAPID-final entails the final configuration of our method.

Method	CWZ (n=46)			Radboud (n=48)			Madabhushi et al. (n=14)		
	Accuracy $\uparrow$	Overlap $\uparrow$	TRE $\downarrow$	Accuracy $\uparrow$	Overlap $\uparrow$	TRE $\downarrow$	Accuracy $\uparrow$	Overlap $\uparrow$	TRE $\downarrow$
SIFT^36^	0.0%	0.90 ± 0.041	15.13 ± 2.17	4.0%	0.86 ± 0.042	13.68 ± 2.01	0.0%	0.94 ± 0.021	6.31 ± 1.95
LightGlue^37^	79.2%	0.98 ± 0.015	1.27 ± 0.63	62.0%	0.96 ± 0.016	1.25 ± 0.78	57.1%	0.97 ± 0.018	0.75 ± 0.49
OmniGlue^27^	60.4%	0.97 ± 0.018	3.90 ± 0.82	50.0%	0.95 ± 0.018	3.57 ± 0.71	57.1%	0.97 ± 0.015	1.66 ± 0.56
RAPID-single-shot	4.2%	0.92 ± 0.045	5.11 ± 3.11	12.0%	0.90 ± 0.046	4.76 ± 3.27	14.3%	0.92 ± 0.056	2.46 ± 1.46
RAPID-no-ransac	91.1%	0.98 ± 0.012	1.23 ± 0.74	84.0%	0.96 ± 0.014	**0.99 ± 0.44**	85.7%	**0.98 ± 0.013**	0.71 ± 0.31
RAPID-overlap	89.6%	**0.98** ± 0.011	**1.07 ± 0.44**	75.5%	0.96 ± 0.014	1.04 ± 0.50	71.4%	0.98 ± 0.013	**0.65 ± 0.26**
RAPID-final	**93.5%**	0.98 ± 0.012	1.27 ± 0.71	**91.7%**	**0.96** ± 0.013	1.14 ± 0.47	**92.9%**	0.97 ± 0.015	0.74 ± 0.33

### Failure mode analysis

**Fig. 6:**
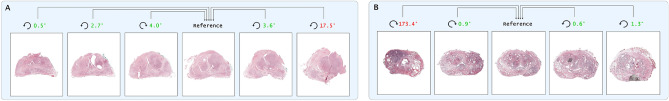
Visualization of the two common failure modes of RAPID, depicted in panel A and B. Panel A shows an example of the failure mode where a slide at the end of the stack had a 17.5 degree rotation mismatch with respect to the reference frame, slightly exceeding the maximum allowed threshold of 15 degrees. Panel B depicts the failure mode where one slide in the stack is misregistered by nearly 180 degrees, leading to an inaccurate reconstruction at this end of the stack. Numbers and arrow direction above each slide indicate deviation from the ground truth rotation with respect to the reference frame, i.e. a slide with a counter-clockwise arrow and 0.5 degree deviation indicates that the slide should be rotated by this magnitude and direction to be fully congruent with the reference frame. Green and red text colour indicate deviations that did or did not meet, respectively, the acceptance threshold of a maximum 15 degree deviation.

Despite RAPID’s robust performance across different external validation sets, there remain instances where the 3D reconstruction is only partially accurate. When pooling these cases (n=8) over the three validation sets, we observe two main categories of errors, which are visualized in Figure 6. The first category, visualized in panel A of Figure 6, entails cases (n=6) where the orientation mismatch between two adjacent WSIs slightly exceeds our empirical acceptance threshold of 15 degrees. On average, this mismatch was 18.6 degrees and tended to occur nearly exclusively (5/6 cases) at the end of the stack, where visual similarity with the adjacent WSI tended to be lower than in the middle of the stack. Interestingly, all these failure cases had only a single WSI in the stack that did not meet our acceptance threshold, indicating that the remainder was accurately reconstructed. Hence, considering this first category as a failure case is primarily an empirical choice, and reconstructions in this category may still be accurate enough depending on the downstream task. The second category (n=2), however, presents a clearer failure mode of RAPID and represents cases with a substantial 90–180 degree rotation mismatch for a single WSI in the stack. In both cases, this orientation mismatch was caused by RAPID not finding the correct 180-degree orientation in the pre-alignment step, which prevented the estimation of a correct rigid transformation later on in the pipeline. Thus, this category highlights the importance of obtaining a rough match in orientation before attempting to match global features and estimating the rigid transform. Lastly, we note that a pooled evaluation of RAPID on all validation sets on the WSI level rather than the patient level demonstrates an orientation accuracy of 98.5% (512/520 WSIs) with a median orientation mismatch of 2.5 degrees. In other words, we observe that all 8/8 failed reconstructions could be salvaged by manually adjusting the rotation of only a single slide in the stack, since the remainder of the stack was accurately reconstructed.

## Discussion

In this work, we have introduced RAPID, a framework for the three-dimensional reconstruction of histopathology specimens from a stack of two-dimensional whole slide images. By leveraging foundation model global features for the reconstruction, our approach enables reconstruction in scenarios where adjacent slides are relatively visually dissimilar, such as sparsely sectioned specimens. Since previous methods were limited to serially sectioned slides, the novel availability of reconstruction from sparsely sectioned specimens enabled by RAPID opens up a wide range of downstream applications, such as automated three-dimensional tissue analysis (i.e. tumour volume estimation) and multimodal data integration (i.e. correlation with 3D pre-operative imaging).

When comparing our approach to the current state-of-the-art reconstruction method VALIS, we find that RAPID performs on par for serially sectioned specimens but outperforms VALIS with increasing performance gaps for increasingly sparser sampled specimens. Although the authors of VALIS acknowledge in their respective paper that their method was not intended for sparse reconstruction tasks^16^, this comparison highlights that the exact nature of the reconstruction task may necessitate a different approach. Specifically, we observe that the general cellular architecture of adjacent WSIs in a sparse sampling strategy varies to such a degree that registration methods attempting to detect similar keypoints and/or fine features are likely to fail due to the absence of good matches and abundance of spurious matches. Indeed, ablation experiments based on pure keypoint matching methods without global feature incorporation followed the same failure modes as VALIS. Hence, incorporating coarser foundation model features that focus on the global tissue architecture seems vital in guiding the reconstruction.

One of the main limitations of our approach is that we formulate the reconstruction task as an iterative pairwise registration problem rather than a direct reconstruction problem. Although this iterative pairwise registration allows for the accurate registration of adjacent WSIs, this method may accumulate errors throughout the stack. For example, a relative orientation mismatch of 5 degrees between adjacent WSIs may be acceptable when this is limited to two adjacent WSIs, but this becomes problematic when a total of *n* adjacent WSI pairs has the same 5-degree orientation mismatch, leading to a $n\times 5$ degree total orientation mismatch between the WSIs at both ends of the reconstructed stack. Although we did not encounter any such error accumulation in any noteworthy number of cases in our multicentre test set, it seems theoretically viable that incorporating the full context of larger anatomical structures (i.e., benign prostatic hyperplasia nodules spanning multiple WSIs) may lead to an improved reconstruction. Hence, a viable future research direction may be to investigate using a broader context with several WSIs to diminish the potential for error accumulation.

Another limitation is that we primarily validated our method on relatively rigid tissue types, where a rigid transformation generally suffices to obtain an accurate three-dimensional reconstruction. However, for tissues that are more prone to deformation, adding a deformable registration component may lead to a reconstruction that is more representative of the original in situ structure of the specimen. However, the main complexity in adding such a deformable component is that a ground truth original tissue shape is generally unavailable. For example, the sparsely sampled breast tissue sections from a (partial) mastectomy could theoretically benefit from a deformable registration due to the flexible nature of the tissue. Still, a ground truth shape of the original (partial) mastectomy specimen is lacking. Hence, the additional value of a deformable registration component would be hard to verify and may lead to overfitting on general shape characteristics of adjacent slides. Nevertheless, there may be scenarios where an additional deformable registration component could be expected to lead to a better reconstruction, and future research could investigate under which conditions such an approach may be warranted.

Lastly, evaluating reconstruction accuracy in sparsely sampled specimens is inherently challenging due to the lack of consistently available landmarks between adjacent slides. While serial sectioning allows the same structure to be identified on adjacent slides and thus supports landmark-based evaluation, the notable slice gap in sparse sampling typically prohibits such an approach. When the slice gap distance exceeds the size of these structures, this structure cannot reliably serve as a landmark since it appears on only a single slide. Furthermore, larger structures may follow a meandering trajectory throughout the specimen, and landmarks relating to such structures thus cannot be expected to share the same XY-coordinate on adjacent slides. Given these challenges, we were unable to evaluate registration error through expert-labelled landmarks for the human prostatectomy datasets, as such landmarks could not reliably be identified. While incorporating such a landmark-based validation strategy is expected to strengthen the validation of our proposed method, this may require the use of strand-shaped fiducial markers in prospective cases.

In conclusion, our method enables the previously unexplored area of generating full-resolution three-dimensional stacks of WSIs from sparsely sampled specimens. We expect that the novel ability to create such three-dimensional reconstructions will open up several avenues in research and the clinic, including improved correlation with pre-operative imaging, quantitative three-dimensional tissue analysis, and improved multimodal data integration.

## Data Availability

We will release a representative example case from our external test set for the reproducibility of our main results. Our terabyte-sized private training and evaluation set is available upon reasonable request. The public datasets used in this study are available from their respective origin described in [21] and [19].

## Code availability

The full source code of the RAPID algorithm is available through https://github.com/computationalpathologygroup/rapid, under the GPL3 license. Additionally, we make a containerized version of RAPID publicly available through the GitHub Container Repository, promoting further adoption of our method.

## References

[1] Gibbons, M. et al. Identification of prostate cancer using multiparametric MR imaging characteristics of prostate tissues referenced to whole mount histopathology. *Magnetic Resonance Imaging* **85**, 251–261. 10.1016/j.mri.2021.10.008 (2022). 34666162 10.1016/j.mri.2021.10.008 PMC9931199

[2] Gibbons, M., Simko, J. P., Carroll, P. R. & Noworolski, S. M. Prostate cancer lesion detection, volume quantification and high-grade cancer differentiation using cancer risk maps derived from multiparametric MRI with histopathology as the reference standard. *Magn. Reson. Imaging* **99**, 48–57. 10.1016/j.mri.2023.01.006 (2023). 36641104 10.1016/j.mri.2023.01.006 PMC11229728

[3] Park, K. J., Kim, M.-H., Kim, J. K. & Cho, K.-S. Characterization and PI-RADS version 2 assessment of prostate cancers missed by prebiopsy 3-T multiparametric MRI: Correlation with whole-mount thin-section histopathology. *Clin. Imaging* **55**, 174–180. 10.1016/j.clinimag.2019.03.004 (2019). 30908991 10.1016/j.clinimag.2019.03.004

[4] Liu, J. T. C. et al. Harnessing non-destructive 3D pathology. *Nat. Biomed. Eng.* **5**, 203–218. 10.1038/s41551-020-00681-x (2021). 33589781 10.1038/s41551-020-00681-x PMC8118147

[5] Liu, J. T., Glaser, A. K., Poudel, C. & Vaughan, J. C. Nondestructive 3D pathology with light-sheet fluorescence microscopy for translational research and clinical assays. *Annu. Rev. Anal. Chem.* **16**, 231–252. 10.1146/annurev-anchem-091222-092734 (2023). 10.1146/annurev-anchem-091222-092734 PMC12829911 36854208

[6] Kiemen, A. L. et al. CODA: Quantitative 3D reconstruction of large tissues at cellular resolution. *Nature Methods* **19**, 1490–1499. 10.1038/s41592-022-01650-9 (2022). 36280719 10.1038/s41592-022-01650-9 PMC10500590

[7] Kiemen, A. L. et al. Tissue clearing and 3D reconstruction of digitized, serially sectioned slides provide novel insights into pancreatic cancer. *Med* **4**, 75–91. 10.1016/j.medj.2022.11.009 (2023). 36773599 10.1016/j.medj.2022.11.009 PMC9922376

[8] Xie, W. et al. Prostate cancer risk stratification via nondestructive 3D pathology with deep learning-assisted gland analysis. *Cancer Res.* **82**, 334–345. 10.1158/0008-5472.CAN-21-2843 (2021). 34853071 10.1158/0008-5472.CAN-21-2843 PMC8803395

[9] Serafin, R. et al. Nondestructive 3D pathology with analysis of nuclear features for prostate cancer risk assessment. *J. Pathol.* **260**, 390–401. 10.1002/path.6090 (2023). 37232213 10.1002/path.6090 PMC10524574

[10] Erion Barner, L. A. et al. Artificial Intelligence-triaged 3-dimensional pathology to improve detection of esophageal neoplasia while reducing pathologist workloads. *Mod. Pathol.* **36**, 100322. 10.1016/j.modpat.2023.100322 (2023). 37657711 10.1016/j.modpat.2023.100322

[11] Jansen, I. et al. Three-dimensional histopathological reconstruction of bladder tumours. *Diagn. Pathol.* **14**, 25. 10.1186/s13000-019-0803-7 (2019). 30922406 10.1186/s13000-019-0803-7 PMC6440143

[12] Song, A. H. et al. Analysis of 3D pathology samples using weakly supervised AI. *Cell* **187**, 2502-2520.e17. 10.1016/j.cell.2024.03.035 (2024). 38729110 10.1016/j.cell.2024.03.035 PMC11168832

[13] Hong, S.-M. et al. Three-dimensional visualization of cleared human pancreas cancer reveals that sustained epithelial-to-mesenchymal transition is not required for venous invasion. *Mod. Pathol.* **33**, 639–647. 10.1038/s41379-019-0409-3 (2020). 31700162 10.1038/s41379-019-0409-3 PMC10548439

[14] Liu, J. T. et al. Engineering the future of 3D pathology. *J. Pathol. Clin. Res.* **10**, e347. 10.1002/cjp2.347 (2023). 37919231 10.1002/cjp2.347 PMC10807588

[15] Kurz, A. et al. 3-dimensional reconstruction from histopathological sections: A systematic review. *Lab. Invest.* **104**, 102049. 10.1016/j.labinv.2024.102049 (2024). 38513977 10.1016/j.labinv.2024.102049

[16] Gatenbee, C. D. et al. Virtual alignment of pathology image series for multi-gigapixel whole slide images. *Nature Communications* **14**, 4502. 10.1038/s41467-023-40218-9 (2023). 37495577 10.1038/s41467-023-40218-9 PMC10372014

[17] Gibson, E. et al. 3D prostate histology image reconstruction: Quantifying the impact of tissue deformation and histology section location. *J. Pathol. Inform.* **4**, 31. 10.4103/2153-3539.120874 (2013). 24392245 10.4103/2153-3539.120874 PMC3869958

[18] Guzmán-Arocho, Y. D. & Collins, L. C. Pragmatic guide to the macroscopic evaluation of breast specimens. *J. Clin. Pathol.* **77**, 204–210. 10.1136/jcp-2023-208833 (2024). 38373781 10.1136/jcp-2023-208833

[19] Madabhushi, A. & Feldman, M. Fused Radiology-Pathology Prostate Dataset (Prostate Fused-MRI-Pathology), 10.7937/k9/TCIA.2016.tlpmr1am (2016).

[20] Chappelow, J., Tomaszewski, J. E., Feldman, M., Shih, N. & Madabhushi, A. HistoStitcher: An interactive program for accurate and rapid reconstruction of digitized whole histological sections from tissue fragments. *Comput. Med. Imaging Graph.* **35**, 557–567. 10.1016/j.compmedimag.2011.01.010 (2011). 21397459 10.1016/j.compmedimag.2011.01.010 PMC3118267

[21] Kartasalo, K. et al. Comparative analysis of tissue reconstruction algorithms for 3D histology. *Bioinformatics* **34**, 3013–3021. 10.1093/bioinformatics/bty210 (2018). 29684099 10.1093/bioinformatics/bty210 PMC6129300

[22] Otsu, N. A threshold selection method from gray-level histograms. *IEEE Trans. Syst. Man Cybern.* **9**, 62–66. 10.1109/TSMC.1979.4310076 (1979).

[23] Reinhard, E., Adhikhmin, M., Gooch, B. & Shirley, P. Color transfer between images. *IEEE Comput. Graph. Appl.* **21**, 34–41. 10.1109/38.946629 (2001).

[24] Oquab, M. et al. DINOv2: Learning Robust Visual Features without Supervision. *ArXiv*10.48550/arXiv.2304.07193. arXiv:2304.07193. (2024).

[25] Song, X., Xu, X. & Yan, P. General Purpose Image Encoder DINOv2 for Medical Image Registration. *ArXiv*10.48550/arXiv.2402.15687 (2024).

[26] Edstedt, J., Sun, Q., Bökman, G., Wadenbäck, M. & Felsberg, M. RoMa: Robust Dense Feature Matching. *ArXiv*10.48550/arXiv.2305.15404 (2023).

[27] Jiang, H., Karpur, A., Cao, B., Huang, Q. & Araujo, A. OmniGlue: Generalizable Feature Matching with Foundation Model Guidance. *ArXiv*10.48550/arXiv.2405.12979 (2024).

[28] Simonyan, K. & Zisserman, A. Very Deep Convolutional Networks for Large-Scale Image Recognition. *ArXiv*10.48550/arXiv.1409.1556 (2015).

[29] Fischler, M. A. & Bolles, R. C. Random sample consensus. *Commun. ACM* **24**, 381–395. 10.1145/358669.358692 (1981).

[30] Cupitt, J. Pyvips (version 2.2.0) (2022).

[31] Saha, A. et al. Artificial intelligence and radiologists in prostate cancer detection on MRI (PI-CAI): An international, paired, non-inferiority, confirmatory study. *The Lancet. Oncology* **25**(7), 879–887. 10.1016/S1470-2045(24)00220-1 (2024). 38876123 10.1016/S1470-2045(24)00220-1 PMC11587881

[32] Reynolds, H. M. et al. Development of a registration framework to validate MRI with histology for prostate focal therapy. *Med. Phys.* **42**, 7078–7089. 10.1118/1.4935343 (2015). 26632061 10.1118/1.4935343

[33] Rusu, M. et al. Registration of presurgical MRI and histopathology images from radical prostatectomy via RAPSODI. *Medical Physics* **47**(9), 4177–4188. 10.1002/mp.14337 (2020). 32564359 10.1002/mp.14337 PMC7586964

[34] Pooli, A. et al. Predicting Pathological Tumor Size in Prostate Cancer Based on Multiparametric Prostate Magnetic Resonance Imaging and Preoperative Findings. *The Journal of Urology* **205**(2), 444–451. 10.1097/JU.0000000000001389 (2021). 33026934 10.1097/JU.0000000000001389

[35] Priester, A. et al. Magnetic Resonance Imaging Underestimation of Prostate Cancer Geometry: Use of Patient Specific Molds to Correlate Images with Whole Mount Pathology. *The Journal of Urology* **197**(2), 320–326. 10.1016/j.juro.2016.07.084 (2017). 27484386 10.1016/j.juro.2016.07.084 PMC5540646

[36] Lowe, D. G. Distinctive image features from scale-invariant keypoints. *Int. J. Comput. Vis.* **60**, 91–110. 10.1023/B:VISI.0000029664.99615.94 (2004).

[37] Lindenberger, P., Sarlin, P.-E. & Pollefeys, M. LightGlue: Local Feature Matching at Light Speed. *ArXiv*10.48550/arXiv.2306.13643 (2023).

